# An empirical study on the influencing mechanism of Chinese university teachers’ wellbeing

**DOI:** 10.3389/fpsyg.2022.970593

**Published:** 2022-10-06

**Authors:** Shuimei Pei, Zhaojun Chen, Xingxia Zhang, Jianpeng Guo

**Affiliations:** ^1^Institute of Education, Xiamen University, Xiamen, China; ^2^College of Humanities, Xiamen Huaxia University, Xiamen, China; ^3^College of Humanities, Yantai Nanshan University, Yantai, China; ^4^Graduate School of International Studies, Hanyang University, Seoul, South Korea

**Keywords:** teachers’ wellbeing, teaching engagement, teaching experience, undergraduate colleges and universities, structural equation modeling

## Abstract

Current studies on teachers’ wellbeing are mainly on lowering stress or burnout. Few studies have noted that faculty wellbeing is related to teaching activities. Teaching engagement and teaching experience are important predictor variables of teachers’ wellbeing, but the internal and external influencing mechanisms of teachers’ wellbeing have not been clearly revealed. Based on the survey data of 7,408 teachers from 271 undergraduate colleges and universities across China, the internal and external influencing mechanisms of teaching engagement and teaching experience on teachers’ wellbeing were investigated through multicluster structural equation modeling. The results were that teachers’ wellbeing was influenced by both teaching engagement and teaching experience. Among teaching engagement, teachers’ pre-class preparation and post-class communication positively influenced teaching experience, but in-class delivery negatively influenced teaching experience. Teaching experience partially mediates the relationship between engagement and wellbeing. At the level of internal influence, the more teachers identify with and feel accomplished by teaching, the more they invest time and energy in teaching; at the level of external influence, the school environment, leadership, and colleague support affect teachers’ wellbeing through the teaching experience. Universities should offer good teaching hardware and software for teachers, provide adequate teaching support, especially encourage teacher-student communication after class, weaken the rigid constraints and controls on teachers’ teaching in class, give teachers enough teaching autonomy, and reduce their teaching burden to inspire teachers to be more actively involved in teaching, improve their teaching experience, and thus enhance their sense of wellbeing.

## Introduction

Over the years studies on teachers’ wellbeing has continued to grow in China and internationally (Harding et al., 2019; Liu and Zheng, 2021; Lei and Hoay, 2022). This growing concern is justified in light of the potential positive or negative outcomes associated with individual teacher and the institution as a whole. The pursuit of happiness is a fundamental human goal. The General Assembly of the United Nations in its resolution 66/281 of 12 July 2012 proclaimed 20 March the International Day of Happiness, recognizing the relevance of happiness and wellbeing as universal goals and aspirations in the lives of human beings around the world (United Nations, 2012). Although people do not have the same perception of happiness, the pursuit of happiness and its realization are always the highest ideals of people. Teachers’ wellbeing is related to teachers’ teaching effectiveness, innovation, as well as to students’ learning. At present, there are many common problems of teachers’ wellbeing in universities: some teachers suffer from high professional pressure, low sense of personal achievement, increased burnout and interpersonal conflicts. The whole society does not realize the importance of faculty wellbeing, and there is a lack of measures and means to effectively improve teachers’ wellbeing. Therefore, it is both theoretically and practically important to explore the influencing mechanisms of university teachers’ wellbeing and propose appropriate improvement strategies.

### The conceptual content of teachers’ wellbeing

The word “happiness,” which is derived from the adjective “happy,” means pleasant, satisfied, joyful, and lucky. The scientifically standardized term for happiness is “wellbeing,” which means “good existence,” a state of health and happiness. However, the meaning of wellbeing is not completely consistent with happy. The former refers to a living state, while the latter is only a psychological state. In the context of Chinese culture, the meaning of “happiness” is: unexpected success or avoiding disaster; happiness; happy; hope; the meaning of “blessing” is opposite to “disaster.”

Wilson (1967) released his masterpiece *Correlates of Self-reported Happiness*, which kicked off the study of wellbeing in the modern sense. Overall, the definition of wellbeing by different scholars shows that some scholars consider wellbeing to be subjective, some consider wellbeing to be objective, and some consider wellbeing to be subject-object unity. The study of subjective wellbeing has emerged in the United States roughly since the 1950s and was introduced to China after the mid-1980s (Xing, 2002). Western scholar Bradburn (1969) mainly believed that the more positive emotions individuals experience and the less negative emotions they experience, then they will get more subjective wellbeing; Diener (1984) believed that subjective wellbeing is a comprehensive psychological indicator of an individual’s quality of life, reflecting the individual’s social functioning and adaptability (Cheng and Furnham, 2004; Wang, 2013) showed through regression analysis that self-esteem is the most important correlated variable of wellbeing. Quite a number of scholars in China believe that subjective wellbeing is inseparable from individual emotional responses and life satisfaction. Zheng et al. (2001) considered subjective wellbeing as a holistic assessment and an important comprehensive psychological indicator of an individual’s quality of life; Shen et al. (2013) proposed that personality is one of the internal factors affecting subjective wellbeing; the empirical study by Yang et al. (2015) showed that family socioeconomic status and multiple dimensions of career values have both unique and interactive effects on medical students’ subjective wellbeing; Zhang et al. (2019) showed that teacher-student relationships positively predicted subjective wellbeing and that teacher-student relationships moderated the first half of the mediating process of parent-child relationships affecting subjective wellbeing through peer relationships.

Wellbeing is people’s perception of their own happiness, an understanding of how well their needs are met, and their judgment of what kind of life state they are in. Pleasure, joy, comfort, satisfaction, etc. are the concrete expressions of wellbeing. Occupational wellbeing was developed along with the deepening of happiness research. Early studies on wellbeing believed that wellbeing was context-independent and had a universal structure. In 1976, work context, occupational context, and organizational context were incorporated into the study of wellbeing (Campbell et al., 1976), including employees’ positive affective and cognitive evaluations of their jobs.

Teacher happiness and teacher wellbeing are two concepts that have emerged one after another in education, and they are restricted to the teaching profession only. Scholars mostly view teacher happiness as the happy state brought by the realization of their professional ideals in teaching activities. In his article *On Teachers’ Happiness*, Professor Tan Chuanbao mentions that teachers’ happiness is a state of existence in which teachers freely realize their professional ideals in their own instructional activities (Tan, 2002). Xiao (2005) also believes that teachers’ professional wellbeing is a continuous and stable feeling of pleasure in the work of teaching by their own standards. According to Tang (2010), elementary school teachers’ professional wellbeing is the cognitive satisfaction and emotional experience arising from their professional activities of teaching and raising children.

Chinese scholars have defined teachers’ professional wellbeing from different perspectives, which can be generally summarized as follows: it is the overall subjective psychological experience obtained by meeting personal needs (survival and development) and the realization of self-worth (Wang, 2009), the fit between individual subjective efforts and objective opportunities and conditions (Cao, 2006), individual’s affirmative value judgment about their workplace environment (Yang, 2009). It is a continuous joyful experience for teachers to have their needs met, their professional aspirations realized, their potential fulfilled, and their own harmonious development (Cai, 2010).

In general, scholars’ concepts of teachers’ wellbeing mainly focus on professional wellbeing, which fully reflects teachers’ happy experience in their professional activities, as well as their ability to generate continuous joyful feeling and overcome negative emotions in the process of education and teaching.

### Constructing a model of university teachers’ wellbeing

There are a variety of research topics related to university faculty wellbeing, including faculty members’ own factors, faculty-student relationships, faculty-colleague relationships, faculty-institutional relationships, workplace environment, academic identity, and faculty mentoring programs and so on.

Based on qualitative interviews, Yuzhu Zhang and Shenghua Jin explored the psychological structure of professional wellbeing of university teachers, compiled the Professional Wellbeing of University Teachers Questionnaire, and analyzed the data using exploratory factor analysis and validated factor analysis methods. The results showed that the professional wellbeing of college teachers is a second-order factor model including six factors: student development, friendly relationship, job satisfaction, job achievement, job autonomy, and value realization (Zhang and Jin, 2013). Other articles found through econometric analysis that the wellbeing of corporate-staffed teachers in private colleges and universities was significantly lower than that of career-staffed teachers, and this finding held true even after controlling for factors such as basic pension insurance, spouse’s status, and working hours. Such status differences greatly frustrates the work motivation of corporate-staffed teachers and hinders the healthy and sustainable development of private colleges and universities. For this reason, there is an urgent need for Chinese government to increase its policy support to treat corporate-staffed teachers in private universities equally and adjust them to career status (Sun and Wang, 2012). One study examined the relationship between university teaching job characteristics and faculty wellbeing by using the JD-R model (Job Demand-Resource Model) as a framework (Han et al., 2019a). The same research team used the JD-R model to examine the associations among challenging job demands, job resources, emotional exhaustion, job engagement and to examine the moderating influence of teacher efficacy as a personal resource at work (Han et al., 2019b). Some studies have examined the relationship between academic identity and faculty wellbeing, particularly on the psychological struggle between the academic identities of “Teachers” and “Researchers.” These psychological struggles are caused by the pressures imposed by the university, which in turn can lead teachers to allocate different amounts of time to different matters depending on their academic identity position (Daryl et al., 2018). Other studies have explored faculty emotions through teaching and interaction with students, providing insight into the emotional experiences of university teachers, and identifying the factors, events, or situations that contribute to faculty emotional pleasantness or unpleasantness (Hagenauer and Volet, 2014). The relationship between managing students’ mental health problems and university faculty wellbeing has been analyzed in the literature, and the “emotional work” poured on students with mental health problems increases the stress of university faculty (Laws and Fiedler, 2012). Resilience can positively predicts the wellbeing of university faculty (Pretsch et al., 2012). The relationship between academic professional identity and university faculty wellbeing, especially for health professional educators, plays an integral role in the wellbeing and professional output of university faculty (Lieff et al., 2012).

Faculty wellbeing, a research hotspot in recent years, involves a fairly wide range of influencing factors. However, as a kind of occupational wellbeing, the professional identity and job characteristics of university teachers clearly qualify the close relationship among teaching engagement, teaching experience, and teacher wellbeing.

Specifically, in the field of higher education, teaching engagement refers to the subjective feelings and actions of teachers’ personal resources in teaching. According to Zhentian Liu, as a guarantee for teachers to release the energy of education and teaching, teaching engagement is actually the teachers’ willingness to teach (Liu, 2013). Sheng (2006) also believes that a teacher’s proactivity and fascination with his or her job is a teacher’s teaching engagement. Zhai (2015) divided teachers’ teaching engagement into time engagement measured by “workload” and emotional engagement measured by “satisfaction.” In contrast to general work, teaching is an educational activity about human beings. Teaching is a complex task, and it is necessary to consider the specific contexts in which university teachers work in a holistic manner. In addition to the input of individual teachers before, during, and after classes, it is also closely related to the overall hardware environment of the university where they work, the support of university leaders and departmental colleagues, and other situational factors.

**FIGURE 1:**
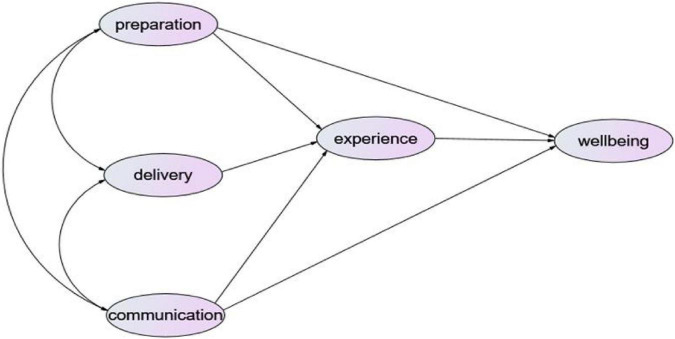
The hypothetical model of the mechanisms influencing teachers’ wellbeing.

Inspired by Donabedian’s Structure, Process, Outcome Model (Donabedian, 1969), we adapted it into the field of higher education. Here Structure refers to how is teaching organized, i.e., teaching engagement consisted of pre-class preparation, in-class delivery and post-class communication; Process refers to teaching experience on autonomy, teaching burden, relationship; Outcome refers to how faculty feel about their wellbeing. And in view of the fact that extant literature have not revealed the influencing mechanism among teaching engagement, teaching experience, and teachers’ wellbeing, based on the above definitions of professional wellbeing, teaching engagement, teaching experience, and incorporating factors at the internal level of individual university teachers and factors at the external level such as university hardware and support from leaders and colleagues, this study proposes the following hypotheses as Figure 1 shows:

Hypothesis I: Teaching engagement directly affects teachers’ wellbeing.

Hypothesis II: Teaching engagement indirectly affects teachers’ wellbeing through mediating the role of teaching experience.

## Materials and methods

### Participants

The research group distributed a questionnaire on the wellbeing of university teachers to undergraduate institutions nationwide through an online survey platform in May, 2020. Faculties completed the survey by logging on to the online survey platform. It took about 10 min to answer all the questions. The questionnaire was submitted only after all questions were answered. All respondents participated in this study voluntarily and gave their informed consent. No participant received any compensation for their involvement in the survey. Assurances regarding the confidentiality of the information provided were given. All participants consented to have their responses used for research purposes. After removing the data such as regular responses, less than 200 s of response time, the remaining 7,408 valid questionnaires were collected from 271 undergraduate higher education institutions in China; 1.42% of the participants work in top universities, 3.21% in top disciplines, 87.99% in general undergraduate universities, and 7.38% in independent colleges. Of the sample, approximately 41.2% were males and 58.8% were females. Faculty in the disciplines of Humanities and social sciences accounted for 51.1%; teachers in the disciplines of science, agriculture, and medicine accounted for 48.9%. The number of those with 0–10 years of teaching experience is 3297, or 44.5%. The number of teachers with 11–20 years of teaching experience was 2282, or 30.8%. The number of those who have taught for 21–30 years is 1089, accounting for 14.7%. The number of those with more than 30 years of teaching experience was 740, or 10.0%. Among the teachers who participated in the survey, 2667 (36.0% of the total) held a doctoral degree, 3971 (53.6% of the total) held a master’s degree, 667 (9.0% of the total) held a bachelor’s degree, and 103 (1.4% of the total) held other degrees.

### Measures

The inventory used in this study was mainly adapted from the Engaged Teachers Scales (Klassen et al., 2013) and The National Questionnaire on Undergraduate Education and Teaching in Chinese Colleges and Universities developed by Guo et al. (2021). The variables of pre-class preparation, in-class delivery, post-class communication, teaching experience, and teacher wellbeing were measured.

The Pre-Class Preparation Inventory consists of three questions that investigate the precourse preparation inputs of university teachers, such as “*I will prepare appropriate teaching materials according to the content of each course.*” The inventory was scored on a four-point Likert scale, with higher scores indicating increasingly adequate teacher preparation input before class.

The In-Class Delivery Inventory consists of four questions that investigate university teachers’ teaching methods, teaching activities, and how to develop students’ competencies. Sample item: “*I will organize teaching activities according to the characteristics of the student*s.” The inventory questions were all scored on a four-point Likert scale, with higher scores indicating more input from university teachers in the teaching process.

The Post-class Communication Inventory consists of four questions that investigate university teachers’ reflection on teaching, correction of assignments, evaluation of students, and communication with students after class. Sample item: “*After class I spend a lot of time in communicating with students.*” The questions were scored on a four-point Likert scale, and higher scores indicated more input from university teachers after class.

The Teaching Experience Inventory consists of three questions that investigate university teachers’ autonomy, teaching burden, and support from leaders and colleagues, including questions such as “*My teaching work load is moderate.*” The questions were scored on a four-point Likert scale, and higher scores indicated better teaching experiences of university teachers.

The Faculty Wellbeing Inventory consists of three questions, investigating the sense of accomplishment and satisfaction that university teachers obtain from teaching, such as the question “*I think it’s great to be a university teacher.*” The questions were scored on a four-point Likert scale, and higher scores indicated higher wellbeing of university teachers.

### Data analysis

In the first stage, confirmatory factor analyses (CFA) were used to assess the reliability and validity of the measurement models using Amos 23.0. In the second stage, the hypothesized relationships between constructs were examined using structural equation modeling (SEM). The internal consistency of each construct was investigated using Cronbach’s alpha and composite reliability (CR). Tests for convergent and discriminant validity were performed to assure the validity of the scales. Convergent validity is confirmed when the indicator factor loadings are statistically significant and exceed the acceptable value of 0.5 for their corresponding constructs and when the average variances extracted (AVEs) for constructs are larger than 0.5. Discriminant validity is assured by the square root of the AVEs being greater than the interconstruct correlations (Fornell and Larcker, 1981). The goodness-of-fit of the CFA and SEM models were evaluated using a number of indices. In addition to the chi-square statistic, the root mean square error of approximation (RMSEA), the confirmatory fit index (CFI), and the non-normed fit index (NNFI) were used as model fit indices in the study. According to the SEM literature (Marsh et al., 1988; Schreiber et al., 2006), the data fit is excellent when RMSEA is less than 0.06, and NNFI and CFI are greater than 0.95, and the data fit is acceptable when RMSEA is less than 0.08, and NNFI and CFI are greater than 0.90. We also performed Harman’s single-factor test to assess the common method bias since we used a single questionnaire method to collect data for all measures in this study (Podsakoff et al., 2003). In the model analysis, we used the bias-corrected bootstrap method to examine the mediating effects of the models. In addition, model applicability and fitness are analyzed by multicluster and multimodel comparisons.

## Results

### Descriptive statistics, correlations, and confirmatory factor analyses

A validated factor analysis containing 17 first-order factors was used to assess the reliability and validity of the measurement models using Amos 23.0. The results showed good fit indices for the measurement model [x^2^(109) = 3606.833, *p* < 0.001, RMSEA = 0.066, NNFI = 0.935, CFI = 0.948]. The loading values for each factor question item were greater than 0.60 and the *t*-values were significant at the 0.01 level. AVE values (average extracted variance for each factor) for all factors were greater than 0.40, and the root of AVE was greater than the correlation coefficient between factors, indicating good discriminant validity. The Cronbach’s alpha coefficient and group reliability for all factors were greater than 0.69, indicating good reliability of the factors. As shown in Table 1, the reliability level is satisfactory and suitable for further analysis (see Table 1).

**TABLE 1:** Correlation matrix, reliability, validity, and descriptive statistics (*N* = 7,408).

Scale	1	2	3	4	5	Cronbach’s α	CR	AVE	CFA loadings range (mean)
1. Pre-class preparation	0.79					0.82	0.83	0.62	0.72–0.83 (0.78)
2. In-class delivery	0.70	0.78				0.86	0.86	0.61	0.72–0.82 (0.78)
3. Post-class communication	0.63	0.77	0.74			0.83	0.83	0.55	0.66–0.78 (0.74)
4. Teaching experience	0.33	0.39	0.42	0.66		0.72	0.69	0.43	0.60–0.69 (0.65)
5. Faculty’s Wellbeing	0.50	0.56	0.56	0.48	0.80	0.84	0.84	0.64	0.70–0.87 (0.79)
Mean	3.76	3.64	3.51	3.32	3.60				
SD	0.40	0.46	0.50	0.63	0.52				

Mean values for scales are total scores divided by the number of items. Possible mean range between 1 and 4 for scales. Lower triangular matrix of columns of the correlation between variables, and the diagonal line is the square root of the average variance extracted (AVE).

Table 1 also shows the mean, standard deviation, and correlation coefficients for each variable. The mean score of 3.76 for university teachers’ preclass preparation is 2.5 points above the theoretical median, indicating that teachers are quite well prepared for class. In-class delivery (*M* = 3.64) and post-class communication (*M* = 3.51) scores indicate that university teachers are more engaged in class and after class. The mean score of 3.32 for teaching experience is also above the theoretical median. The score (*M* = 3.60) indicates that university teachers have a higher overall wellbeing. Overall, the descriptive results indicate that university teachers scored higher in teaching engagement (including preparation, delivery, and post-class communication) than in teaching experience.

The correlation matrix in Table 1 shows the expected significant correlations between subscales. Moderate correlations were found between all variables except teaching experience. Teaching experience was moderately correlated with in-class delivery and wellbeing, and showed weak correlations with other variables. Based on the results of the correlation analysis, the hypothesized relationships between the variables could be further tested subsequently using structural equation modeling.

### Structural equation modeling analysis

SEM was applied to investigate the hypothesized relationship among preclass preparation, in-class delivery, post-class communication, teaching experience, and teacher wellbeing in accordance with the theoretical assumptions described in the “Introduction” section. The goodness-of-fit of the estimated model was evaluated by the following indicators: root mean square error of approximation (RMSEA), Tucker Lewis index (TLI/NNFI) and comparative fit index (CFI). The cutoff values for good-fitting models were as follows: CFI and NNFI > 0.90 and RMSEA < 0.09 (Hu and Bentler, 1999). This model had good fit indices [x^2^(110) = 3114.950, *p* < 0.001, RMSEA = 0.061, NNFI = 0.944, and CFI = 0.955]. The hypothesis that the mechanisms influencing university teacher wellbeing were supported. Pre-class preparation and post-class communication positively influenced the teaching experience, and in-class delivery negatively influenced the teaching experience. University teachers’ wellbeing was positively predicted by pre-class preparation, in-class delivery, post-class communication, and teaching experience.

**FIGURE 2:**
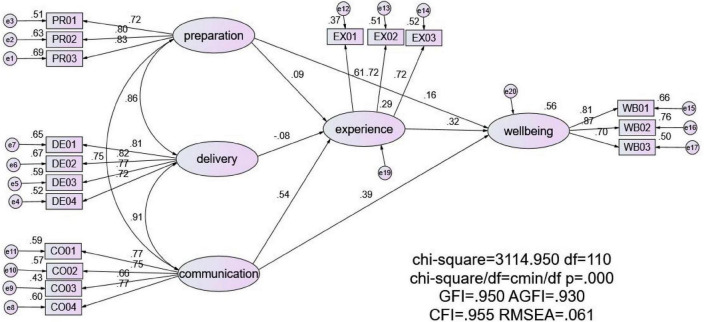
Standardized structural relations among variables (*N* = 7,408).

In Figure 2, all paths with standardized coefficients are statistically significant at the 0.001 level. Controlled demographic variables are omitted in the figure to maintain the clarity of the model. The variables in the model explained 29% of the variance in teaching experience and 56% of the variance in teacher wellbeing.

### Mediating effect

In addition to the direct predictive effect in the model, the mediating effect results are revealed. A significant partial mediating effect of teaching experience between preclass preparation and teacher wellbeing (β = 0.031, 95% CI: 0.016–0.045, *p* < 0.001). A significant partial mediating effect of teaching experience between in-class delivery and teacher wellbeing (β = 0.074, 95% CI: 0.058–0.089, *p* < 0.001). A significant partial mediating effect of teaching experience between post-class communication and teacher wellbeing (β = 0.099, 95% CI: 0.083–0.114, *p* < 0.001). A significant partial mediating effect of teaching experience between in-class delivery and teacher wellbeing (β = 0.074, 95% CI: 0.058–0.089, *p* < 0.001). A significant partial mediating effect of teaching experience between post-class communication and teacher wellbeing (β = 0.099, 95% CI: 0.083–0.114, *p* < 0.001).

We performed a group path analysis by imposing a gender and faculty ranks constraints on the path estimates. Of the 7408 participants, approximately 41.2% were males and 58.8% were females. Professors accounted for 11.6%, associate professors were 34.3%, assistant professors were 44.1% and others were 10.0%. In China, promotion to the next higher rank in a faculty classification is based on a faculty member’s academic and professional qualifications and achievements in the categories of teaching, scholarship and service. Chinese universities usually requires faculties to teach at least 5 years in the current rank position before promoting to the next higher rank. So in the vast majority of cases, the older a university faculty member is, the higher his or her rank. The goodness-of-fit for the constrained models was found to be as good as that for the unconstrained models (ΔIFI < 0.005, ΔNFI < 0.005, ΔRFI < 0.005, ΔTLI < 0.005), indicating that the effect estimates did not differ by gender and faculty ranks. This also supports the reasonableness of the hypothesized model in this study.

## Discussion

This study integrated the components of teaching engagement and teaching experience to construct a model of the influence of university teachers’ wellbeing, and explored the relationship between teaching engagement (including pre-class preparation, in-class delivery, and post-class communication), teaching experience, and teacher wellbeing in a more comprehensive manner. The results of the structural equation model supported the hypothesis of this study that teacher wellbeing is influenced by teaching engagement and teaching experience. Pre-class preparation and post-class communication positively predicted teaching experience; the more prepared university teachers were before class and the more they interacted with students after class, the more positive their teaching experience was. In-class delivery negatively predicts teaching experience. Teaching experience positively predicted teacher wellbeing. There was a significant partial mediating effect of teaching experience between teaching engagement and teacher wellbeing. The results of the multicluster analysis further supported the relationship between the effects of teacher wellbeing at the individual teacher level and the university level.

First, at the internal faculty level, the teaching engagement of carefully designing the course syllabus, spending sufficient time preparing for the class, and preparing appropriate teaching materials for each course all positively predicted the teaching experience. Adequate preparation helps faculty build confidence in their teaching. Confidence is a fundamental psychological quality and a strong motivator and guarantor of what people can do. Adequate preparation helps teachers to be “prepared” for any changes or “surprises” in the teaching process and to “improvise” so that they can have a good teaching experience of “playing to their heart’s content” and “being at ease.” Confidence in teaching does not come out of thin air; it can only be acquired through continuous accumulation and learning. Both individual faculty members and the university community need to focus on lesson planning and standardize it. In today’s information age, with information readily available on the Internet, it is important to be wary of the prevalence of “electronic lesson planning,” where lesson plans for several semesters of a course can simply be copied and pasted. However, this kind of preparation is different from the correct preparation because there is no study of teaching standards and no design of teaching methods. Consequently it will prevent teachers from thinking about and practicing instructional design, which is detrimental to the improvement of teaching effectiveness and teachers’ professional growth. Faculty should pay attention to preclass preparation, the university administration also should standardize lesson planning, so that faculty can be prepared to carry out effective teaching while positively predicting teachers’ wellbeing.

Second, giving teachers enough autonomy over their teaching in universities positively predicts teachers’ wellbeing, which is consistent with Higgins and Kram (2001). Teaching is teacher-led and university teachers should be able to dictate their own teaching activities and the elements of teaching involved. The academic administration of higher education institutions should not be too detailed and rigid in the formulation of teaching plans, arrangement of teaching contents, selection of teaching materials, selection of teaching equipment, and organization of teaching activities inside and outside the classroom for specific courses, and they should not introduce overly rigid policies. Under the premise of ensuring the primacy of teachers’ morality and style, what and how to teach at the level of specific lectures, “returning” the university classrooms to faculties, giving them enough teaching autonomy is conducive to various teaching experiments and reforms, enhancing teachers’ teaching experience and indirectly enhancing teachers’ wellbeing, thus taking comprehensive teaching reform as the grasp to further strengthen undergraduate teaching. The university will further strengthen the quality of undergraduate teaching with the comprehensive teaching reform.

Third, after the expansion of colleges and universities, especially when China enters the stage of popularization of higher education, many colleges and universities across the country have a mismatch between the scale of expansion of teaching resources such as teachers and the scale of expansion of students. The phenomenon of “teacher-student ratio” has been highlighted, which often leads to an increase in the teaching tasks of university teachers. Faculties’ teaching tasks are often aggravated, especially by the simple adoption of quantitative assessment indexes, which specify the number of class hours for teachers with different titles and different types of classes. In addition to the “explicit” teaching hours on the podium, there are also “implicit” teaching burdens such as preparing new lectures, changing assignments, and making comments. This study found a negative effect between teaching-load-based in-class delivery and teacher experience, which is consistent with the findings of Demerouti et al. (2001). The JD-R model suggests that when individuals require high levels of effort to maintain performance and protect themselves from fatigue, it can lead to health problems such as fatigue, low energy, and work exertion. Similarly, Han et al. (2019a) found a significant negative association between work resources and emotional exhaustion among university teachers. High levels of work resources can alleviate teachers’ work stress under demanding working conditions. Cordes and Dougherty (1993) also showed a significant relationship between social support and emotional exhaustion. Therefore, in the top-level design of universities, it is suggested that the “explicit” and “implicit” contributions of the special work nature of university teachers’ teaching should be taken into consideration, and the assessment of university teachers should be given some flexibility. Different indicators should be set for teachers of different titles and disciplines. In addition to adjusting rigid assessment indexes, faculty development centers and departmental management of colleges and universities should also pay attention to humanistic care for teachers, shape a good working atmosphere with leaders’ care and support, colleagues’ mutual help and assistance, take appropriate ways to enhance faculties’ self-efficacy and master psychological debugging methods, establish a positive mindset and reasonable professional expectations, improve faculties’ wellbeing, avoid university teachers’ burnout and even depression or any extreme conditions.

## Implications for educational practice

This study uses empirical survey data to reveal the internal and external mechanisms that affect university teachers’ wellbeing at the individual and collective levels, and the findings have theoretical and practical implications. This study attempts to subdivide teaching engagement into three parts: preparation before class, delivery during class, and communication after class, then integrates external teaching experience factors, proposes a theoretical model between teaching engagement, teaching experience, and teachers’ wellbeing, and validates it with large sample data. The results of the study help to explore the factors influencing faculties’ wellbeing and reveal its mechanism. In particular, in view of the existence of multiple theoretical bases and models in the current research on teachers’ wellbeing, unlike some other theoretical frameworks that focus on teachers’ salary, research pressure, and burnout, the theoretical framework proposed in this study is based on teachers’ individual input before, during, and after classes, as well as on the collective university experience in terms of hardware, leadership and colleague support, teaching burden, which can be used as a basis for scholars’ subsequent research on teachers’ professional wellbeing in higher education. It can be used as a basis for scholars to follow up on the professional wellbeing of university teachers.

The findings of the present study have important practical implications. The mechanisms influencing faculties’ wellbeing revealed in this study can provide a reference for improving teaching quality and enhancing teachers’ wellbeing in China. The results indicate that teaching inputs (including pre-class preparation, in-class delivery, and post-class communication) play an important role in teachers’ professional wellbeing. Pre-class preparation and post-class communication directly and positively affected teaching experience, especially the path coefficient between post-class communication and teaching experience was 0.55 (*p* < 0.001), indicating that high-quality post-class communication significantly enhanced teaching experience. In the management of higher education, it is necessary to emphasize the preparation of the syllabus and the design of lesson plans, but it is also necessary to fill the gap of “communication between teachers and students after class.” After the popularization of higher education in China, the number of students has increased dramatically, the student-teacher ratio of many applied undergraduate colleges and universities remains high, and large class teaching is common. Thanks to the development of information technology, various instant messaging softwares have changed the way of communication between teachers and students in colleges and universities after class, but “key-to-key” cannot be fully equated with “face-to–to-face” after all. By changing the evaluation system and increasing the proportion of after-school communication in teachers’ evaluation, higher education institutions can change the concept that teachers reduce their work to classroom teaching and cultivate their responsibility of educating people. As a concrete measure, universities can set up an office hour system, in which teachers arrange a fixed time in a fixed place every week to answer students’ questions according to their own schedules. The content of communication is not limited to the course, but can be extended to examination, competition, academic planning, preparation for graduate school, etc. Colleges and universities can also establish a corresponding “appointment-consultation” system through campus networks and other information platforms to facilitate personalized and in-depth communication between teachers and students. In addition, the results of this study show that in-class teaching has a negative impact on teaching experience, which indicates that teachers in colleges and universities are challenged to analyze the learning situation, control the classroom, and develop students’ critical thinking and logical reasoning, thus creating pressure to negatively affect the teaching experience. In addition to the various types of regular training conducted at the university level through the Faculty Development Center, each department works in course groups to analyze the teaching content and student characteristics, then discuss how to choose reasonable teaching methods and how to organize teaching activities accordingly. In the regular class, there are various means to enhance students’ critical thinking and logical reasoning ability. First of all, the teaching process should be brave to break the teacher’s “one-word” model, and encourage students to dare to question and fully express their own views; secondly, the teaching design should intentionally create problematic situations to stimulate students’ curiosity, which is conducive to cultivating students’ originality of thinking; third, the content of the course should be chosen with an emphasis on logical reasoning, helping students to develop the habit of understanding things from multiple perspectives, such as “seeking differences in similarities” and “seeking similarities in differences,” so that they can express themselves clearly. The teachers’ experience will be enhanced through increased input in preparation, delivery, and communication at the individual and collective levels. The better the teaching experience is for university teachers, the higher their wellbeing will be.

## Limitations and directions for future research

There are several limitations that need to be addressed and directions for future research that need to be pointed out. Firstly, although this study constructed the model of “teaching engagement-teaching experience- teachers’ wellbeing,” it only investigated university teachers’ teaching engagement before, during, and after classes. It did not analyze teachers’ engagement in research and social services, which may also affect the wellbeing of the whole group of university teachers. In addition, this study mainly measured the relevant variables through scales, and although scales, a self-reporting method, are commonly used to measure wellbeing, they are, after all, susceptible to the influence of university teachers’ subjective factors. Moreover, this study used cross-sectional survey data to study the one-way predictive relationship between variables, which is essentially an exploratory study, and it is more difficult to truly consider the causal predictive relationship between each variable. There should be two-way interactions between these variables, for example, teaching engagement affects teaching experience, and in turn teaching experience affects teaching engagement; teaching experience affects wellbeing, and in turn wellbeing affects teaching experience. Future research should further adopt longitudinal tracking data to better determine the sequential causal relationships between variables and build a more scientific model of university teachers’ wellbeing. Moreover, future research could consider using objective indicators such as interviews and observations for measurement. For example, by collecting university teachers’ performance data, interviewing faculty themselves to see teachers’ wellbeing in their own minds, interviewing university leaders and faculties’ families to see teachers’ wellbeing in the eyes of others.

## Data availability statement

The raw data supporting the conclusions of this article will be made available by the authors, without undue reservation.

## Ethics statement

The studies involving human participants were reviewed and approved by the Xiamen University Ethics Committee. The participants in this study volunteered to fill in the online questionnaire anonymously and they could quit anytime. The participants’ answers were just for this academic research and all information was kept confidential.

## Author contributions

SP: literature review, methodology, formal analysis, implication, and writing. ZC: constructing the model and hypotheses and reviewing. XZ: discussion and editing. JG: conception of the work, investigation, and interpretation. All authors have read and agreed to the published version of the manuscript.
